# Is it possible? Predicting complications and morbidity in surgical patients on clopidogrel therapy with Thrombelastography Platelet Mapping

**DOI:** 10.1007/s10195-013-0267-6

**Published:** 2013-08-30

**Authors:** Rashida Callender, Alfonso Altamirano, Tiffney Tezino, Evan G. Pivalizza, Davide Cattano

**Affiliations:** Department of Anesthesiology, The University of Texas Medical School at Houston, 6431 Fannin Street, MSB 5.020, P.O. Box 20708, Houston, TX 77030 USA

Dear Editor,

We read with great interest the article by Hossain et al., in April 2013 “Is discontinuation of clopidogrel necessary for intracapsular hip fracture surgery? Analysis of 102 hemiarthroplasties”. An increasing number of patients presenting for anesthesia are taking clopidogrel and an even greater number are on combination antiplatelet therapies, potentially increasing the risk of intraoperative and perioperative bleeding.

Hossain et al. [1] found no increase in complications or transfusions in patients with perioperative clopidogrel and those without. Collyer et al. [2], conducted a similar study on 114 patients receiving regular clopidogrel therapy and presenting for urgent hip fracture surgery. While an increased risk of requiring blood transfusion during or after surgery was noted in patients off clopidogrel for only 1 day, no major complications were reported.

It is our opinion that correlation between platelet function and responsiveness to clopidogrel is of paramount importance. There is significant individuality in patient responsiveness to clopidogrel, suggesting that an individualized, evidence-based approach is needed to assess the risk of adverse outcomes in patients receiving regular clopidogrel therapy. We suggest the use of the Thrombelastograph ^®^ Platelet Mapping^™^ (TEG-PM) assay for preoperative assessment of platelet ADP receptor inhibition. Collyer et al. [2, 3], provided insight to weak and unpredictable responses to clopidogrel therapy and the risk of acute coronary syndrome in patients off clopidogrel for more than 4 days. This emphasizes the importance of measuring platelet function to determine residual clopidogrel therapy.

**Fig. 1:**
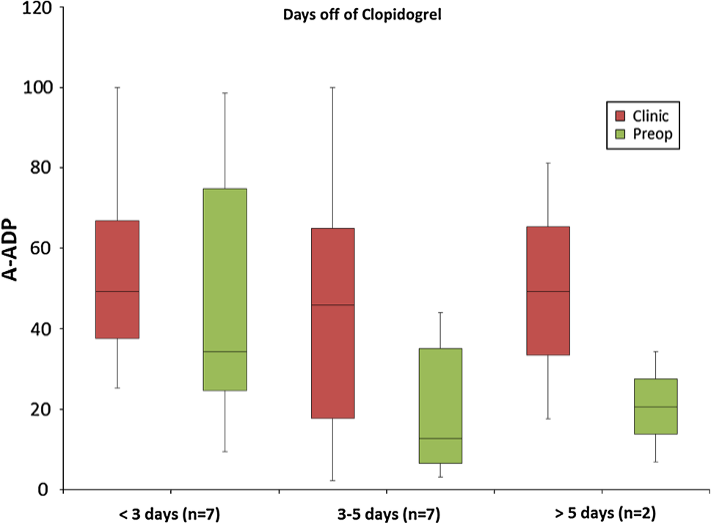
Whisker plot distribution comparing anesthesia clinic and preoperative percent (%) ADP inhibition in 16 patients based on days off clopidogrel. Anesthesia clinic values are presented in *red* while preoperative values are presented in *green*

We assessed the ability of TEG-PM to detect preoperative platelet function secondary to clopidogrel and/or aspirin therapy [4]. In an expansion of the study, we assessed preoperative platelet function in 131 patients based on days off of clopidogrel and/or aspirin on the day of surgery. Of the 131, 16 patients were followed continuously from the time of their preoperative anesthesia clinic visit to the day of surgery (Fig. 1; Table 1).

**Table 1:** Anesthesia clinic and preoperative percent (%) platelet inhibition (mean ± SD) in 16 patients separated into three groups by their days off of clopidogrel (<3, 3–5 and >5 days)

Days off of clopidogrel	*n*	Anesthesia clinic % ADP inhibition		Preoperative %ADP inhibition	
		Mean	Median	Mean	Median
<3 days	7	54.8 ± 25.8	49.3 (37.6, 66.8)	48.7 ± 35.8	34.4 (24.7, 74.8)
3–5 days	7	44.8 ± 34.9	46.0 (17.8, 64.9)	20.4 ± 17.7	12.7 (6.6, 35.1)
>5 days	2	49.4 ± 45.0	49.4 (33.5, 65.3)	20.6 ± 19.4	20.6 (13.8, 27.5)
Total patients	16	49.7 ± 30.2	47.7 (23.7, 73.9)	32.8 ± 29.6	26.4 (8.8, 44.0)

Data are presented as mean ± SD, and median (1st and 3rd quartile)

*SD* standard deviation, *ADP* adenosine diphosphate

Two important findings were: (1) a trend towards platelet recovery by day 5 off clopidogrel, (2) 60 % of the patient population were not effectively inhibited while on therapy [4]. In a prospective observational study of 59 patients, Collyer et al. [3] also observed a decline in % ADP inhibition with longer interruption of clopidogrel as well as inconsistent efficacy of clopidogrel to inhibit platelet function. Our findings suggest that a preoperative TEG-PM assay might be a feasible approach to objectively evaluate effects of clopidogrel during the perioperative period, guide drug management and avoid adverse outcomes related to hypercoagulability and thrombosis as well as increased bleeding risk. We recommend a larger study correlating TEG-PM values with the incidence of complications and transfusions in older patients presenting for urgent hip fracture surgery.
